# Reduced up-regulation of the nitric oxide pathway and impaired endothelial and smooth muscle functions in the female type 2 diabetic goto-kakizaki rat heart

**DOI:** 10.1186/s12986-016-0157-z

**Published:** 2017-01-13

**Authors:** Martine Desrois, Carole Lan, Jamileh Movassat, Monique Bernard

**Affiliations:** 1 Aix-Marseille Université, CNRS, CRMBM, Marseille, France; 2 Université Paris-Diderot, CNRS, UMR 8251, Laboratoire de Biologie et Pathologie du Pancréas Endocrine (B2PE), Unité BFA, Paris, France; 3 Centre de Résonance Magnétique Biologique et Médicale (CRMBM), UMR n°7339, Aix-Marseille Université, CNRS, Faculté de Medecine, 27 Bd Jean Moulin, Marseille Cedex 05, 13385 France

**Keywords:** Type 2 diabetic heart, Gender differences, Cardiac function, Energy metabolism, Endothelial function

## Abstract

**Background:**

Type 2 diabetes is associated with greater relative risk of cardiovascular diseases in women than in men, which is not well understood. Consequently, we have investigated if male and female displayed differences in cardiac function, energy metabolism, and endothelial function which could contribute to increased cardiovascular complications in type 2 diabetic female.

**Methods:**

Male and female Control and type 2 diabetic Goto-Kakizaki (GK) isolated rat hearts were perfused during 28 min with a physiological buffer before freeze-clamping for biochemical assays. High energy phosphate compounds and intracellular pH were followed using ^31^P magnetic resonance spectroscopy with simultaneous measurement of contractile function. Nitric oxide (NO) pathway and endothelium-dependent and independent vasodilatations were measured as indexes of endothelial function. Results were analyzed via two-way ANOVA, *p <* 0.05 was considered as statistically significant.

**Results:**

Myocardial function was impaired in male and female diabetic versus Control groups (*p <* 0.05) without modification of energy metabolism. Coronary flow was decreased in both diabetic versus Control groups but to a higher extent in female GK versus male GK rat hearts (*p <* 0.05). NO production was up-regulated in diabetic groups but to a less extent in female GK rat hearts (*p <* 0.05). Endothelium-dependent and independent vasodilatations were impaired in female GK rat compared with male GK (*p <* 0.05) and female Control (*p <* 0.05) rat hearts.

**Conclusions:**

We reported here an endothelial damage characterized by a reduced up-regulation of the NO pathway and impaired endothelial and smooth muscle functions, and coronary flow rates in the female GK rat hearts while energy metabolism was normal. Whether these results are related to the higher risk of cardiovascular complications among type 2 diabetic female needs to be further elicited in the future.

**Electronic supplementary material:**

The online version of this article (doi:10.1186/s12986-016-0157-z) contains supplementary material, which is available to authorized users.

## Background

Cardiovascular diseases (CVDs) are the major causes of morbidity and mortality in patients with diabetes mellitus. CVDs are long-term complications of type 2 diabetes mellitus, with a two-fold increased risk of heart failure and greater mortality after myocardial infarction [1]. Diabetic women have a greater relative risk for CVDs than diabetic men, and newly diagnosed diabetic women showed higher relative risk for cardiovascular death than diabetic men in the large DECODE study [2]. Women with type 2 diabetes may undergo even more adverse changes in coagulation, inflammation and vascular function than men [3, 4]. Stronger associations have been reported between insulin resistance/metabolic syndrome and inflammation and endothelial dysfunction in women than in men [5]. Interestingly, it is proposed that women have to undergo greater metabolic deterioration than men to develop type 2 diabetes and as such many insulin resistance risk factors must change to a greater extent [6]. However, few studies have explored the sex differences in the emerging risk factor profile in individuals with or without type 2 diabetes. Consequently, we have investigated if male and female without ischemic insult already displayed differences in cardiac function, energy metabolism, and endothelial function which could contribute to increased cardiovascular complications in type 2 diabetic female. This study was performed in male and female type 2 Goto-Kakizaki (GK) rats.

Six independent genetic loci are responsible for the defects in glucose and insulin metabolism in the Goto-Kakizaki (GK) rat, a highly inbred strain derived from outbred, glucose-intolerant Wistar rats that spontaneously develop type 2 diabetes within the first few weeks of age [7]. The GK is one of the best-characterized animal models of spontaneous type 2 diabetes mellitus [8]. In this model, cardiac insulin resistance was associated with impaired insulin signaling pathway [9]. We have previously shown greater hypertrophy, lower insulin-stimulated glucose uptake rates and increased ischemic injury in the female compared with the male GK rat hearts [10]. In addition, modification of the NO pathway was involved in increased susceptibility of the type 2 diabetic GK rat heart to ischemic injury [11]. Significantly, this model allows one to study the effect of diabetes on the heart without other complications such as obesity.

Here, the effect of gender on type 2 diabetic heart was assessed in male and female Control Wistar and GK isolated rat hearts perfused with a physiological buffer as described previously [11]. High-energy phosphates and intracellular pH were measured during the experimental time course by ^31^P magnetic resonance spectroscopy with simultaneous measurement of contractile function. Energy compounds and oxidative stress in cardiac tissues were evaluated by High Performance Liquid Chromatography (HPLC). Myocardial tissue content of creatine kinase and lactate dehydrogenase were also used as markers of cellular damage. Total nitrate concentration as well as expression of endothelial nitric oxide synthase, AKT and Phospho-Akt were determined as markers of the NO pathway. Endothelium-dependent and independent vasodilatations were also measured in separate experiments in order to assess, respectively, endothelial and smooth muscle functions. A preliminary form of this study has been published as an abstract [12].

## Methods

### Animals

Age-matched Control Wistar (male, *n =* 19; female, *n =* 24) and Goto-Kakizaki rats (GK/Par subline [8]) (male, *n =* 22; female, *n =* 23) (7–8 mo, weight 265–512 g) were used in the experiments. Animals were fed ad libitum with a commercial pelleted chow (diet 113, SAFE, Augy, France).

### Heart perfusion and experimental protocol

Rats were anaesthetized by intraperitoneal injection of 35 mg/kg pentobarbital sodium. After removal of the heart, blood samples were taken from the chest cavity and immediately centrifuged, and the supernatant was kept on ice for determination of plasma glucose and free fatty acids (FFAs). Hearts were cannulated and perfused in the Langendorff mode at constant pressure as described previously [11]. The end-diastolic pressure was set to 10 mmHg for all groups at the beginning of perfusion. Left ventricular developed pressure and heart rate were monitored as previously reported [13]. The rate pressure product (RPP) (product of left ventricular developed pressure and heart rate) was used as an index of cardiac function [11]. Coronary flow was measured by time collection of the coronary venous effluent.

### Experimental protocol for ^31^P magnetic resonance spectroscopy and biochemical analyses

Control (male *n =* 10, female *n =* 14) and GK (male *n =* 13, female *n =* 12) hearts were perfused during 4 min with non-recirculating phosphate-free Krebs-Henseleit bicarbonate buffer which had the following composition (mM): NaCl (118), KCl (4.7), CaCl_2_ (1.75), MgSO_4_ (1.2), ethylenediaminetetraacetate tetrasodium (0.5), NaHCO_3_ (25) and glucose (11). After stabilization, hearts were perfused for 28 min with a physiological recirculating Krebs-Henseleit buffer containing 0.4 mM palmitate, 3% albumin, 11 mM glucose, 3U/L insulin, 0.8 mM lactate and 0.2 mM pyruvate. The perfusion solutions were gassed with a mixture of 95% oxygen and 5% carbon dioxide to give a pH of 7.4 and the temperature was maintained at 37 °C.

### ^31^P magnetic resonance spectroscopy on isolated perfused rat heart

Perfused rat hearts were placed in a 20-mm magnetic resonance sample tube and inserted into a ^31^P probe that was seated in the bore of a superconducting wide-bore (89-mm) 4.7 Tesla magnet (Oxford Instruments, Oxford, UK) interfaced with a Bruker-Nicolet WP-200 spectrometer (Bruker, Karlsruhe, Germany). ^31^P spectra were obtained by the accumulation of 328 free induction decay signals acquired during 4 min (flip angle, 45°; time resolutio*n =* 0.7 s; spectral width, 4500 Hz; 2048 data points). Prior to Fourier transformation, the free induction decay was multiplied by an exponential function which generated a 20-Hz line broadening. The appropriate conditions for acquiring ^31^P magnetic resonance spectra, the quantification of phosphorus metabolites and the determination of intracellular pH have been detailed previously [11]. Quantification of the signal integrals was carried out using an external reference containing an aqueous solution of 0.6 M phenylphosphonic acid. A series of eight ^31^P NMR spectra were recorded during the perfusion protocol.

### Collection of data

Heart function and ^31^P magnetic resonance spectra were simultaneously monitored during the perfusion protocol. Blood samples were collected immediately after excising the heart. For biochemical analyses, hearts were rapidly freeze-clamped with a Wollenberger clamp precooled in liquid nitrogen at the end of the experimental protocol and kept at −80 °C before analysis.

### Biochemical analyses in plasma

Plasma glucose and free fatty acids (FFAs) were determined as described previously [11].

### Biochemical analyses in freeze-clamped hearts

#### Creatine, phosphocreatine, adenine nucleotides and malondialdehyde (MDA)

Determination of creatine, phosphocreatine, adenine nucleotides and MDA was performed as described previously [11]. MDA was used to evaluate lipid peroxidation as a measure of oxidative stress [14].

#### Lactate dehydrogenase (LDH) and creatine kinase (CK) activities and water content

LDH, CK and water content were measured as previously described [15].

#### NO pathway

Total nitrate concentration as well as total protein expression of Akt, Phospho-Akt and endothelial NOS (eNOS) were measured to assess the NO pathway.

##### Total nitrate concentration (NOx)

NOx was determined according to the method described by Cross et al. [16].

##### Protein expression of Akt, Phospho-Akt and eNOS

A portion of cardiac tissue was homogenized as described by Ye et al. [17]. Protein samples (50 μg for Akt, Phospho-Akt and 90 μg for eNOS) were run with 4–20% Tris–HCl ready gel (Thermo Scientific) or 6% SDS-PAGE, respectively and transferred to pure nitrocellulose membrane. The membranes were incubated overnight at 4 °C with primary antibodies against eNOS (1/1000, Becton Dickinson (DB) Transduction Laboratories, USA), Akt (1/1000, Cell Signaling Technology, Inc.), Phospho-Akt (Ser473)(1/1000, Cell Signaling Technology, Inc.) and β-actin (1/5000, Sigma) and secondarily with HRP-conjugated anti-mouse or anti-rabbit antibodies (Santa Cruz Biotechnology, Inc.). The immunoblots were developed using ECL^TM^ Western Blotting Detection Reagent (Amersham^TM^). The protein signals were quantified using the MicroChemi 4.2 system (DNR Bio-Imaging Systems Ltd., Israel). The intensity of each protein signal was normalized to the corresponding β-actin stain signal. Data are expressed as ratios between the protein and the corresponding β-actin signal density.

### Endothelium dependent and independent vasodilatations

In separate experiments, endothelium-dependent and independent vasodilatations were measured using 5-hydroxytryptamine (5-HT, 10^−7^ M) and papaverine (5*10^−6^ M) to assess endothelial and smooth muscle functions respectively, as previously described [15] in Control (male *n =* 9; female, *n =* 10) and GK (male, *n =* 9; female, *n =* 11) isolated perfused rat hearts with Krebs-Henseleit buffer. The 5-HT and papaverine hydrochloride were dissolved in the buffer to give the desired concentration and were obtained from Sigma Chemical Co. (St. Louis; Missouri). The coronary flow was recorded during the perfusion with the Krebs-Henseleit buffer and during the infusion of 5-HT or papaverine. The increase in coronary flow during drug infusion was calculated and expressed as a percentage of the basal value.

### Expression of results and statistical analyses

Data are expressed as means ± SEM. Function and ^31^P magnetic resonance spectroscopy data are presented as absolute values. Significant differences between groups were determined using two-way ANOVA with repeated measures over time for the time-dependent variables (function and ^31^P magnetic resonance spectroscopy data) followed by Bonferroni post-hoc test with Graphpad Prism software (Graphpad prism 5.0, La Jolla, CA). For biochemical data, the effects of time and group were analyzed with two-way ANOVA followed by Bonferroni post-hoc test. Unpaired Student’s *t*-test was used for other parameters. A p value of less than or equal to 0.05 was considered to indicate significant difference.

## Results

### Physiological parameters of male and female Control and GK rats

Plasma glucose was 67% and 69% higher in male and female GK rats versus their respective Controls (Table 1, *p <* 0.05). Plasma FFAs were similar in the four groups (Table 1). Heart to body weight ratio was 23% higher in male GK compared with male Control (Table 1, *p <* 0.0001) due to a significantly lower body weight of male GK compared with male Control (*p <* 0.05) and similar heart wet weights in both groups. In female GK, heart to body weight ratio was 25% higher compared with female Control with a similar body weight in both groups but a significantly higher heart weight in female GK versus female Control (*p <* 0.0001).

**Table 1:** Physiological parameters in Control (male *n =* 10, female *n =* 14) and GK (male *n =* 13, female *n =* 12) rats

	Control		GK	
	Male	Female	Male	Female
Plasma glucose mM	10.7 ± 0.3	10.0 ± 0.3	17.9 ± 0.6*	16.9 ± 0.4*
Plasma FFAs mM	0.27 ± 0.03	0.27 ± 0.05	0.28 ± 0.04	0.26 ± 0.04
Body weight g	512 ± 13	265 ± 7^†^	411 ± 4*	270 ± 5^‡^
Heart weight g	1.52 ± 0.03	0.88 ± 0.04^†^	1.51 ± 0.02	1.12 ± 0.03*^‡^
Ratio Heart/Body weight *1000	2.98 ± 0.05	3.34 ± 0.14^†^	3.66 ± 0.02*	4.17 ± 0.08*^‡^

*versus respective Controls, *p <* 0.0001. ^†^versus Male Control, *p <* 0.05. ^‡^versus Male GK, *p <* 0.0001

### Gender effect on myocardial function

**Fig. 1:**
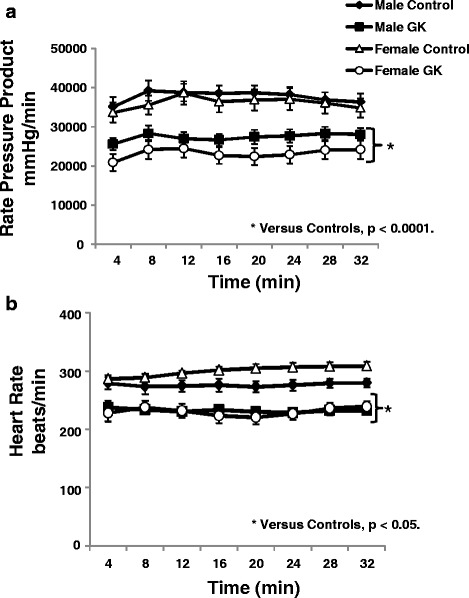
Rate pressure product (RPP, mmHg/min) (**a**) and Heart Rate (mmHg) (**b**) in Control (male *n =* 10, female *n =* 14) and GK (male *n =* 13, female *n =* 12) rat hearts. Results are means ± SEM. * versus respective Controls, *p <* 0.0001

Myocardial function as represented by the rate pressure product (RPP), was significantly decreased in male and female diabetic animals compared with the respective Controls (*p <* 0.0001, Fig. 1a) due to a lower heart rate in male and female GK rat hearts (*p <* 0.0001, Fig. 1b) vs Controls. End diastolic pressure (EDP, mmHg) was not different between groups (Control, male 10 ± 2, female 7 ± 2; GK male 8 ± 1, female 9 ± 2).

**Fig. 2:**
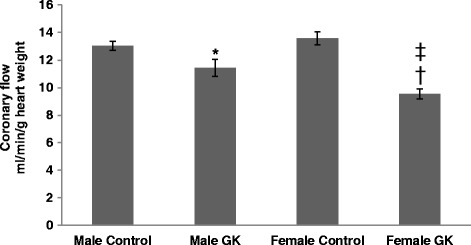
Coronary flow (CF), expressed in ml/min/g heart weight, in Control (male *n =* 10, female *n =* 14) and GK (male *n =* 13, female *n =* 12) rat hearts. Results are means ± SEM. * *p =* 0.0420 and † *p <* 0.0001, versus respective Controls; ‡ *p =* 0.0137, versus male GK rat hearts

Coronary flow (CF) in Control and diabetic rat hearts was shown in Fig. 2. CF was decreased in male and female GK rat hearts compared with their respective Controls (*p =* 0.0420 and *p <* 0.0001 respectively). Interestingly, CF was significantly lower in female GK compared with male GK rat hearts (*p =* 0.0137). No difference was shown between male and female Control rat hearts.

### Gender effect on energy metabolism and intracellular pH (pHi)

**Fig. 3:**
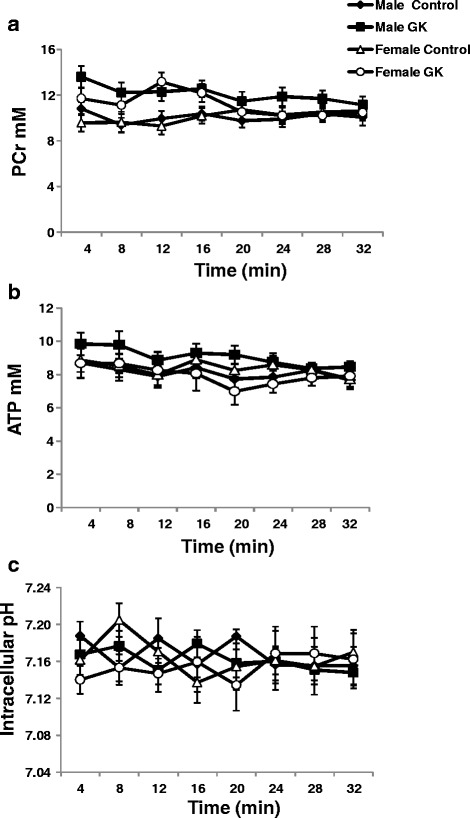
Kinetics of phosphocreatine (PCr) (**a**), adenosine triphosphate (ATP) (**b**) and intracellular pH (pHi) (**c**) in Control (male *n =* 10, female *n =* 14) and GK (male *n =* 13, female *n =* 12) rat hearts, measured by ^31^P magnetic resonance spectroscopy. Results are expressed in mM except pHi, and are means ± SEM

Kinetics of PCr (A), ATP (B) and intracellular pH (C) as measured by ^31^P magnetic resonance spectroscopy are shown in Fig. 3. No significant differences were found in PCr and ATP contents in male and female diabetic and Control rat hearts. pHi was the same in all groups without any significant differences (Fig. 3c). Kinetics of phosphomonoesters (PME) and inorganic phosphate (Pi) were shown in an (Additional file 1: Figure S3d and S3e, respectively). PME and Pi were similar in all groups.

### Gender effect on creatine, adenine nucleotide compounds and oxidative stress

The total pool of creatine (creatine and phosphocreatine) was similar in all groups (Table 2). No significant difference was found in total adenine nucleotides and adenylate energy charge between male and female control and diabetic rat hearts (Table 2). MDA content (expressed in μmol/g protein), as an index of oxidative stress, was not different in male and female GK rat hearts (0.06 ± 0.01 and 0.05 ± 0.00 respectively) compared with male and female Control rat hearts (0.06 ± 0.00 and 0.06 ± 0.00).

**Table 2:** Total pool of creatine, total adenine nucleotides and adenylate energy charge in Control (male *n =* 10, female *n =* 14) and GK (male *n =* 13, female *n =* 12) rat hearts

	Control		GK	
	Male	Female	Male	Female
Total pool of creatine μmol/g protein	94.3 ± 2.8	90.7 ± 4.6	90.9 ± 3.4	94.5 ± 1.6
TAN μmol/g protein	40.9 ± 1.1	37.5 ± 1.3	38.2 ± 1.6	39.9 ± 1.3
AEC	0.83 ± 0.008	0.84 ± 0.007	0.84 ± 0.004	0.85 ± 0.008

*TAN* total adenine nucleotides (ATP + ADP + AMP), *AEC* adenylate energy charge ((ATP + 0.5ADP)/(ATP + ADP + AMP) * 10)

### Gender effect on cellular damage and water content

LDH and CK activities (expressed in U/mg protein) were similar in male (2.39 ± 0.10 and 5.96 ± 0.30) and female (2.32 ± 0.11 and 5.62 ± 0.28) Controls, and male (2.40 ± 0.17 and 6.02 ± 0.35) and female (2.40 ± 0.23 and 5.89 ± 0.27) GK rat hearts. Water content, expressed as a percentage, was not significantly different in male and female Control (83.65 ± 0.75 and 83.10 ± 1.80) and GK (83.62 ± 1.79 and 84.18 ± 0.91) rat hearts.

### Gender effect on NO pathway

#### Total nitrate concentration (NOx)

Tissue NOx content in male and female Control and GK rats was shown in Table 3. We found an increased NOx in both diabetic groups compared with their respective Controls, indicating up-regulation of the NO pathway, but to a less extent in female GK rat hearts with a lower NOx content in female compared with male GK rat hearts (*p =* 0.0004).

**Table 3:** Total nitrate concentration (NOx) in Control (male *n =* 10, female *n =* 14) and GK (male *n =* 13, female *n =* 12) rat hearts

	Control		GK	
	Male	Female	Male	Female
NOx nmol/mg protein	0.18 ± 0.02	0.19 ± 0.01	0.39 ± 0.03*	0.24 ± 0.01^†‡^

*p < 0.0001 versus Male Control rat hearts ^†^ *p =* 0.0113 versus Female Control rat hearts

^‡^ *p =* 0.0004 versus Male GK rat hearts

#### Protein expression of Akt, Phospho-Akt and eNOS

**Fig. 4:**
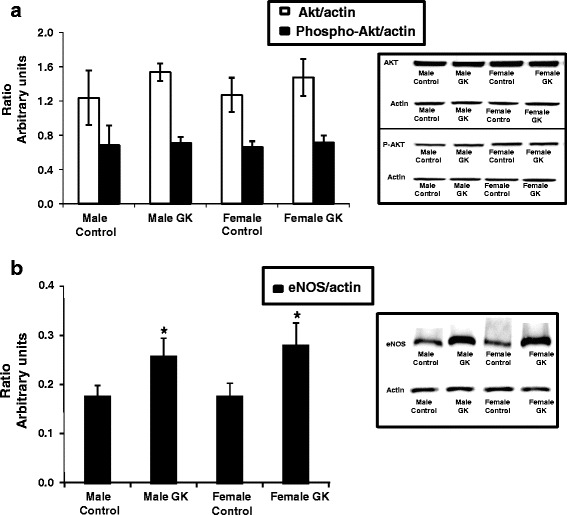
Protein expression of Akt, Phospho-Akt (**a**) and eNOS (**b**) in Control (male *n =* 10, female *n =* 14) and GK (male *n =* 13, female *n =* 12) rat hearts. Protein expression were measured by western blot assay and results are expressed as a ratio relatively to actin protein content and are means ± SEM. * versus respective Controls, *p <* 0.05

Protein expressions of Akt, Phospho-Akt (A) and eNOS (B) were given as ratios relative to actin protein content and were shown in Fig. 4. We found similar protein expressions of Akt and Phospho-Akt in the four groups (Fig. 4a). Interestingly, eNOS expression (Fig. 4b) was significantly increased in both male and female GK rat hearts compared with their respective Controls without any effect of gender (*p <* 0.05).

### Gender effect on endothelial and smooth muscle functions

Endothelium-dependent and independent vasodilatations were shown in Table 4. Endothelium-dependent and independent vasodilatations were not different in male Control and GK rat hearts. By contrast, endothelium-dependent and independent vasodilatations were significantly impaired in female GK compared with male GK (*p <* 0.05) and female Control (*p <* 0.05) rat hearts.

**Table 4:** Endothelium-dependent and independent vasodilatations in Control (male *n =* 9, female *n =* 10) and GK (male *n =* 9, female *n =* 11) rat hearts

	Control		GK	
	Male	Female	Male	Female
5-HT %	32.6 ± 3.0	33.9 ± 3.3	30.5 ± 2.1	19.9 ± 2.6*^†^
Papaverine %	28.0 ± 3.7	29.4 ± 3.2	32.3 ± 4.4	19.2 ± 2.5*^†^

*p < 0.05 versus Male GK rat hearts. ^†^ *p <* 0.05 versus Female Control rat hearts

## Discussion

The aim of the study was to investigate if male and female without ischemic injury displayed differences in cardiac function, energy metabolism, and endothelial function which could contribute to increased cardiovascular complications in type 2 diabetic female. Myocardial function was impaired similarly in both male and female diabetic GK rats. Cardiac energy metabolism was normal in both diabetic groups compared with their respective Controls. Conversely, coronary flow was decreased in both diabetic groups but to a higher level in female GK rat hearts. Total nitrate concentration was up-regulated in both diabetic groups but to a less extent in female GK rat hearts. eNOS/actine was similarly increased in both male and female GK groups without modification of Akt pathway in all groups. Endothelium-dependent and independent vasodilatations were impaired only in female GK rat hearts. Together, these results could be related to higher risk of cardiovascular complications in type 2 diabetic female.

It is known than non-diabetic men are at more risk of developing cardiovascular disease than non-diabetic women. Interestingly, here the non-diabetic male and female rats do not show any difference in coronary flow or endothelial function which could be explained by the lack of stress conditions such as ischaemic insult. However the relative risk of cardiovascular disease incidence and mortality associated with type 2 diabetes compared with non-diabetes is stronger in women than in men [18, 19]. There are well characterized differences in traditional risk factors among diabetic men and women although these do not fully explain the gender differences observed. The reasons why diabetes in women increases the relative risk of CHD more than in men compared with their non-diabetic counterpart is not clear, but a possible explanation may be that diabetes has a greater adverse effect on CVD risk factors in women than in men. Previous studies have shown differences in lipid abnormalities to be more pronounced between diabetic and non-diabetic women than between diabetic and nondiabetic men [20] but this dyslipidemia appears insufficient to explain the differences in clinical risk [21]. Wannamethee et al. [6] have reported that the greater adverse influence of diabetes per se on abdominal adiposity and insulin resistance, and down-stream blood pressure, lipids (low HDL-cholesterol), endothelial dysfunction (t-PA), and systemic inflammation (WBC) in women compared with men may contribute to their greater relative risk of coronary heart disease. Interestingly, we have also previously reported higher insulin resistance in the female than in the male GK rat hearts [10]. Another possible explanation may be due to a need for women to undergo much larger metabolic perturbances to transit from non-diabetes to diabetes, ie in general women may have to “deteriorate” more to get diabetes, they need to put on more weight, and deteriorate their insulin sensitivity and related risk factors to a greater extent than men [6]. On the other hand, the reason for the greater relative risk of CVD associated with diabetes in women compared with men may be also due to the difference in the treatment of cardiovascular heart disease risk factors between men and women or gender response to therapy [22, 23].

Endothelial dysfunction is an early sign of diabetic vascular disease and reduced endothelium-dependent vasorelaxation (EDV) to vasodilators is generally used as a reproducible parameter to investigate the endothelial function under various pathological conditions. Here, the endothelial function was evaluated by a panel of markers, including the NO pathway (NOx production and AKT, Phospho-AKT and eNOS expression) combined to endothelial and smooth muscle vasodilatations and to the measurement of the coronary flow. NO production was increased in both GK rat groups but was less pronounced in female GK rat hearts. Total eNOS protein expression was similarly increased in both diabetic groups as reported before [11, 24] without any effect of gender. AKT protein expression and phosphorylation were similar in diabetic groups indicating that AKT did not play a major role in gender effect on the NO regulation. It has been hypothesized that upregulation of eNOS in diabetes was a consequence of the enhanced oxidative stress induced by hyperglycemia [24–26] and inactivation of NO by the production of reactive oxygen intermediates. MDA production, an index of oxidative stress, was similar in both male and female Control and GK rat hearts suggesting a normal oxidative stress, by contrast to our previous study showing increased MDA content in older male GK rat hearts [11]. Consequently, it would be interesting to evaluate both reactive oxygen species production and the anti-oxidant defence in male and female GK rat hearts in order to accurately rule out a role of oxidative stress on NO modulation in male and female type 2 diabetic GK rat hearts. Finally, measuring the state of eNOS phosphorylation, which is critical for NO synthesis, should be performed to further investigate the NO pathway. Lower NO up-regulation in female GK hearts is difficult to explain. Decreased NO availability in female GK rat hearts may be linked to a decrease in NOS activity due to increased NOS uncoupling [24] and/or impaired intracellular BH4/BH2 [27].

Decreased coronary flow and lower NOx content in the female diabetic rat hearts were associated with impaired endothelium-dependent and independent vasodilatations in the female GK rat hearts. By contrast, up-regulation of the NO pathway in the male GK rat hearts was probably involved in normal endothelial and smooth muscle functions but, nevertheless, this was insufficient for maintaining a normal coronary flow. Interestingly, we have reported higher decrease in basal coronary flow with higher increase in NOx production in male Control and GK older animals (9–14 months) [11]. Impaired endothelium-dependent vasorelaxation (EDV) has been observed in both type 1 and type 2 diabetes [28], wherever some studies have shown enhanced EDV in diabetes [29]. Interestingly, Kobayashi et al. [30] reported enhanced acetylcholine-induced relaxation and impaired norepinephrine-induced contraction, due to NO overproduction via eNOS and increased α_2D_-adrenoceptor expression in early-stage GK rats. Impaired acetylcholine-induced relaxation in later-stage GK rats is due to reductions in both NO production and NO responsiveness. Conflicting data were also obtained when responses to vasoconstricting agents were studied [30, 31]. The reason for these discrepancies is not clear. However, the duration of the disease, among other factors, may play an important role in the extent of the alteration of vascular reactivity to vasodilating or vasoconstricting agents in diabetes [32]. Zhang et al. [33] demonstrated that Ach-induced relaxations were significantly impaired in mesenteric arteries from both male and female diabetic rats at one and eight weeks. Interestingly, the extent of impairment was significantly greater in diabetic females than in diabetic males at eight weeks suggesting a shift away from a putative endothelium-derived hyperpolarizing factor (EDHF) towards a greater reliance on NO. Several other reports [34, 35] suggest that hyperglycemia and diabetes affect male and female vascular beds differently. Clinically, these differences reveal a stronger association between CVD and diabetes in women than in men [36, 37]. Interestingly, Goel et al. [34, 35] reported a predisposition of female rabbit aorta compared with male rabbit aorta toward impairment of endothelium-dependent vasodilation under hyperglycemic conditions, possibly via activation of PKCβ and superoxide production. Gender differences in sex hormones may be also one explanation for the differences in NO production/release in GK rats. Vascular strips from female rats were found to release more NO in response to acetylcholine than vascular strips from male rats [38]. These data suggest that estrogen may directly stimulate NO production/release in women. Conversely, the predominant male sex hormone testosterone (or other androgens) may cause decreased NO production/release, as suggested by Herman [39]. Interestingly Al-Mulla et al. [40] reported reduced estrogen and increased testosterone levels in the female GK rats and the possible roles of these hormones in inflammatory processes involved in wound healing impairment in type 2 diabetes. The independent contributions of estrogens and androgens to the control of endothelial function in normal and pathophysiological states, especially in type 2 diabetes, remain to be fully elucidated.

Myocardial function was significantly decreased in female GK rats and to the same extent than in the male GK rats. As suggested before [11], impaired cardiac function was probably related to a significantly lower heart rate in both GK rat hearts compared with their Controls, possibly caused by hyperglycemia which alters excitation-contraction coupling by lengthening the period of mechanical relaxation [41]. On the other hand, we have previously shown a 38% decreased protein level of IRS1, one of the major insulin-signaling component, in male GK rat hearts [9], which could be also involved in impaired cardiac function in diabetic rats as reported by Qi et al. [42]. Interestingly, Soliman et al. [43] reported that the RhoA/ROCK pathway contributes to contractile dysfunction in diabetic heart at least in part by sustaining PKCβ2 activation, iNOS activation and ROS production via a positive feedback loop that requires an intact cytoskeleton. Mitochondrial dysfunction could be also involved in impaired cardiac function in both GK rat heart groups as reported recently in high-fat diet mice [44]. On the other hand, peroxisome proliferator-activated receptors (PPARs) may also play a role in functional and metabolic abnormalities of the type 2 diabetic GK rat heart [45]. However, here cardiac energy metabolism was normal in female GK rat hearts and similar to the male GK rat heart suggesting normal mitochondrial respiration in GK rat hearts. We have also previously reported that increased susceptibility of older male type 2 diabetic GK rat heart to ischemic injury was not associated with impaired energy metabolism [11]. By contrast, reduced myocardial phosphocreatine/ATP ratio, indicating impaired high energy phosphate-metabolism and energy deficit [46, 47] has been reported in human diabetic cardiomyopathy. However Diamant et al. [48] found a decreased PCr/ATP in type 2 diabetic patients but did not confirm this finding in a subsequent study with a group of well-controlled uncomplicated type 2 diabetic patients [49], probably due to differences in patient characteristics.

### Limitations

The experiments were conducted here on isolated perfused hearts. In this model, we do not have the interactions with the other organs and with the whole body physiology and metabolism, which has both advantages and limitations. The advantage of this model is to be able to study the intrinsic properties of the heart alone without the interactions with the other organs and whole physiology.

On the other hand, studying the heart in vivo using magnetic resonance imaging or echocardiography has an additional value by taking in account the whole physiology. In accordance with the results of the present study, using multiparametric magnetic resonance imaging, we have previously shown that adult female GK rats had defective myocardial blood flow associated with altered left ventricular function in vivo [50], which is consistent with the ex vivo results reported here.

## Conclusion

Here, we studied hemodynamic function, energy metabolism, cellular integrity and endothelial function in male and female Control and GK rat without ischemic insult in order to check if gender differences already exist at basal state which could explain increased cardiovascular complications in type 2 diabetic female. We reported an endothelial damage characterized by reduced up-regulation of the NO pathway combined with impaired endothelial and smooth muscle functions and coronary flow rates in female diabetic rat hearts while energy metabolism was normal. Whether these results and involved molecular mechanisms are related to the higher risk of cardiovascular complications among type 2 diabetic female waits to be further elicited in the future.

## Additional file

Additional file 1: Figure S3. Kinetics of phosphomonoesters (PME) (D) and inorganic phosphate (Pi) (E) in Control (male *n =* 10, female *n =* 14) and GK (male *n =* 13, female *n =* 12) rat hearts, measured by ^31^P magnetic resonance spectroscopy. Results are expressed in mM and are means ± SEM. (DOCX 70 kb)

## Abbreviations

CK: Creatine kinase
eNOS: Endothelial nitric oxide synthase
LDH: Lactate dehydrogenase
NO: Nitric oxide
