# Supplementary material for: Reduced up-regulation of the nitric oxide pathway and impaired endothelial and smooth muscle functions in the female type 2 diabetic goto-kakizaki rat heart
Source: Nutr Metab (Lond). 2017 Jan 13;14:6. doi: 10.1186/s12986-016-0157-z (PMC5237314; doi:10.1186/s12986-016-0157-z)
Supplement: Additional file 1: Figure S3. — Kinetics of phosphomonoesters (PME) (D) and inorganic phosphate (Pi) (E) in Control (male n = 10, female n = 14) and GK (male n = 13, female n = 12) rat hearts, measured by 31P magnetic resonance spectroscopy. Results are expressed in mM and are means ± SEM. (DOCX 70 kb) [file 12986_2016_157_MOESM1_ESM.docx]

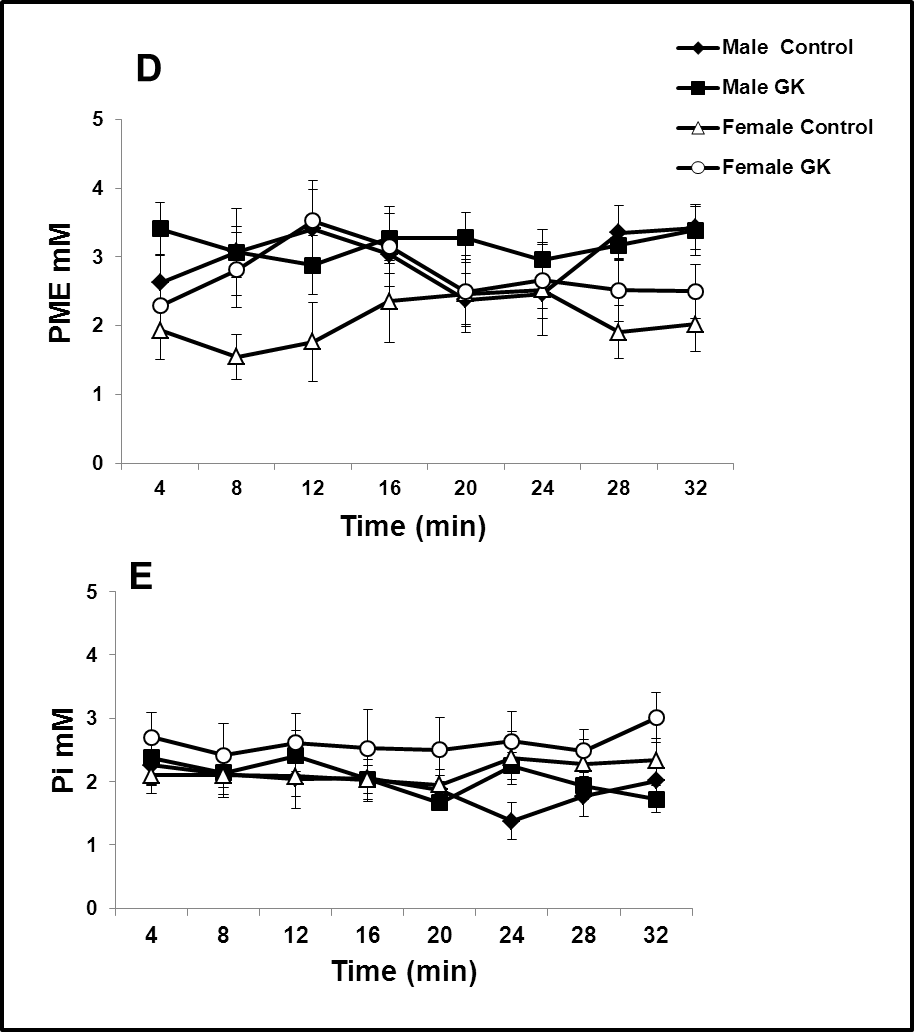


**Figure 3** : Kinetics of phosphomonoesters (PME) (D) and inorganic phosphate (Pi) (E) in Control (male n = 10, female n = 14) and GK (male n = 13, female n = 12) rat hearts, measured by ^31^P magnetic resonance spectroscopy. Results are expressed in mM and are means ± SEM.
